# To capture the child’s interest - nurses experiences of ‘Saga stories in health talks’

**DOI:** 10.1186/s12912-023-01661-7

**Published:** 2024-01-02

**Authors:** Camilla Collan, Lina Dahl, Maria Henström, Christine Delisle Nyström, Marie Löf, Susanne Andermo

**Affiliations:** 1 Department of Neurobiology, Care Sciences and Society, Division of Nursing, Karolinska Institutet, Huddinge, 141 83 Sweden; 2 Department of Biosciences and Nutrition, Karolinska Institutet, Neo, Huddinge, 141 83 Sweden; 3 Department of Health, Medicine and Caring Sciences, Division of Society and Health, Linköping University, Linköping, 581 83 Sweden; 4 Department of Physical Activity and Health, The Swedish School of Sport and Health Sciences, Stockholm, 114 33 Sweden

**Keywords:** Health promotion, Nurse, Children, Families, Lifestyle behaviours, Child health care, Illustrative material

## Abstract

**Background:**

As unhealthy lifestyle habits have been found to be established early in life and often track into adulthood, early preventive initiatives are important. **‘**Saga Stories in health talks’ is a newly developed material that is intended to be used as a support for nurses at child health care (CHC) centers in their health talks with children and parents in Sweden. The aim of this study is to explore how CHC nurses experience the usability of the ‘Saga Stories in health talks’ material.

**Methods:**

This study used a qualitative design. The material **‘**Saga Stories in health talks’ was tested by 33 CHC nurses working in 11 CHC centers in three regions in Sweden. All CHC nurses were invited to participate in the interviews and 17 agreed. The interviews were transcribed and analysed using content analysis.

**Results:**

Three categories and eight sub-categories emerged. The categories were: (1) *An appreciated tool suitable for health talks*, (2) *Illustrations to capture children’s interest in the conversation with families*, and (3) *Barriers and facilitators.* Saga Stories in health talks’ was experienced by the CHC nurses as an appreciated tool with content highly relevant to what should be discussed during the health talks. The CHC nurses described the material as well-designed with illustrations that helped them capture the child’s interest and increase their participation, while still involving the parents. Support from colleagues, the researchers, and managers were seen as important facilitators. Challenges included structural factors such as how and when to best use the material, especially concerning that the 4-year visit contained many other mandatory parts.

**Conclusions:**

This pilot study show that the material **‘**Saga Stories in health talks’ was highly appreciated by CHC nurses and facilitated their health talks with families in CHC. Important aspects with the material were the relevant content and the focus on healthy living habits, as well as the child friendly illustrations. These findings can be used when similar material is developed to facilitate health talks with families in other contexts. Our results also highlight the importance to adjust the implementation of a new material with already established practice and routines.

**Supplementary Information:**

The online version contains supplementary material available at 10.1186/s12912-023-01661-7.

## Background

The World Health Organization (WHO) has identified childhood obesity as one of the greatest challenges for public health in the 21st century [1]. In Sweden, one in ten 4-year-olds have overweight or obesity [2]. As lifestyle habits, such as diet and physical activity have been found to be established early in life and often tracks into adulthood [3–6], preventive initiatives targeted at young children and their parents should be prioritized to improve public health.

Swedish child health care (CHC) has a central and important role in health promotion work through their regular contact with families. CHC is free of charge and almost all children in Sweden attend the child health services national program [7, 8]. The program consists of routine visits throughout the first five years of life, and the families can receive more frequent visits when needed [9]. In addition to monitoring children’s growth, development and health, an important component of the national program is dedicated to health promotion work, which includes the promotion of healthy lifestyle habits such as diet, physical activity, and sedentary behavior. As part of the health promotive work, at routine visits all families receive health talks with CHC nurses based on the families’ need and preferences [9]. Thus, CHC nurses have a key role in health promotion initiatives and support for families with children aged 0–5 years.

Nurse-led interventions to prevent overweight and obesity in child health care include different forms of education to families and parents about healthy lifestyle behaviors, often with motivational interviewing [10]. Several studies have explored barriers and facilitators in health promotion work directed to families with young children, specifically concerning weight-related discussions [11–15]. Helle et al. [15] address that public health nurses play a pivotal role in nutritional guidance for parents of infants, but that they need more support and updated knowledge for this task. Previous research have also shown that nurses in Sweden experienced insufficient skills and tools to manage children with overweight and obesity [14]. A review of qualitative studies concerning health care professionals’ experiences of barriers and facilitators to discuss child weight with parents showed that the most common barriers included a perceived lack of knowledge among the health care professionals and fear of parental reactions [11]. In a qualitative study with CHC nurses in Sweden, Sjunnestrand et al. [13] describe that nurses are reluctant to address children´s weight with parents. This reluctance is due to several factors; including a wish to preserve the parents trust, a perceived lack of knowledge on their own communication skills and a lack of knowledge on how to provide further health care guidance to the parents. In another study, describing nurses´ experiences of child-centered health dialogues with children with overweight and their caregivers, Castor et al. [12] showed that these dialogues could be challenging on an emotional level. Such challenges and barriers in the health care encounters could lead to a reluctance to discuss certain topics in health care encounters with parents. Bohman et al. [16] have addressed these aspects, when they found that dietary and physical activity behaviors are given infrequent attention at the routine visits in CHC. Apart from nurses’ communicative skills and use of different communicative techniques, nurses use a variety of methods and materials to facilitate their work with health talks directed to families in CHC.

A central intention with the use of visual material in health talks is to facilitate the discussion and strengthen the relation between the nurse and the child [17]. Thus, to facilitate CHC nurses’ work in CHC centers, supportive tools, methods, and resources are being developed to be used as a compliment to routine care. ‘Saga Stories in health talks’ is newly developed material that is intended to be used as support for CHC nurses at CHC centers in their health talks with children and parents. However, before being implemented within CHC the usability and effectiveness of these tools need to be determined. Specifically, the perspectives of CHC nurses are critically important to understand the usability of Saga Stories in health talks. Such understanding is essential in the process of implementation of such materials. Thus, the aim of this study is to explore how CHC nurses experience the usability of the material **‘**Saga Stories in health talks’.

## Methods

### Design

The study has a qualitative design, with individual interviews with CHC nurses that were analyzed with content analysis, to get a better understanding of the CHC nurses’ experiences from using the ‘Saga Stories in health talks’ material [18, 19]. This pilot study is part of a larger study where the material **‘**Saga Stories in health talks’ is explored and evaluated at CHC in Sweden. The COnsolidated criteria for REporting Qualitative research (COREQ) checklist were used to report the research process, see Appendix 1.

### Saga stories in health talks

**Fig. 1:**
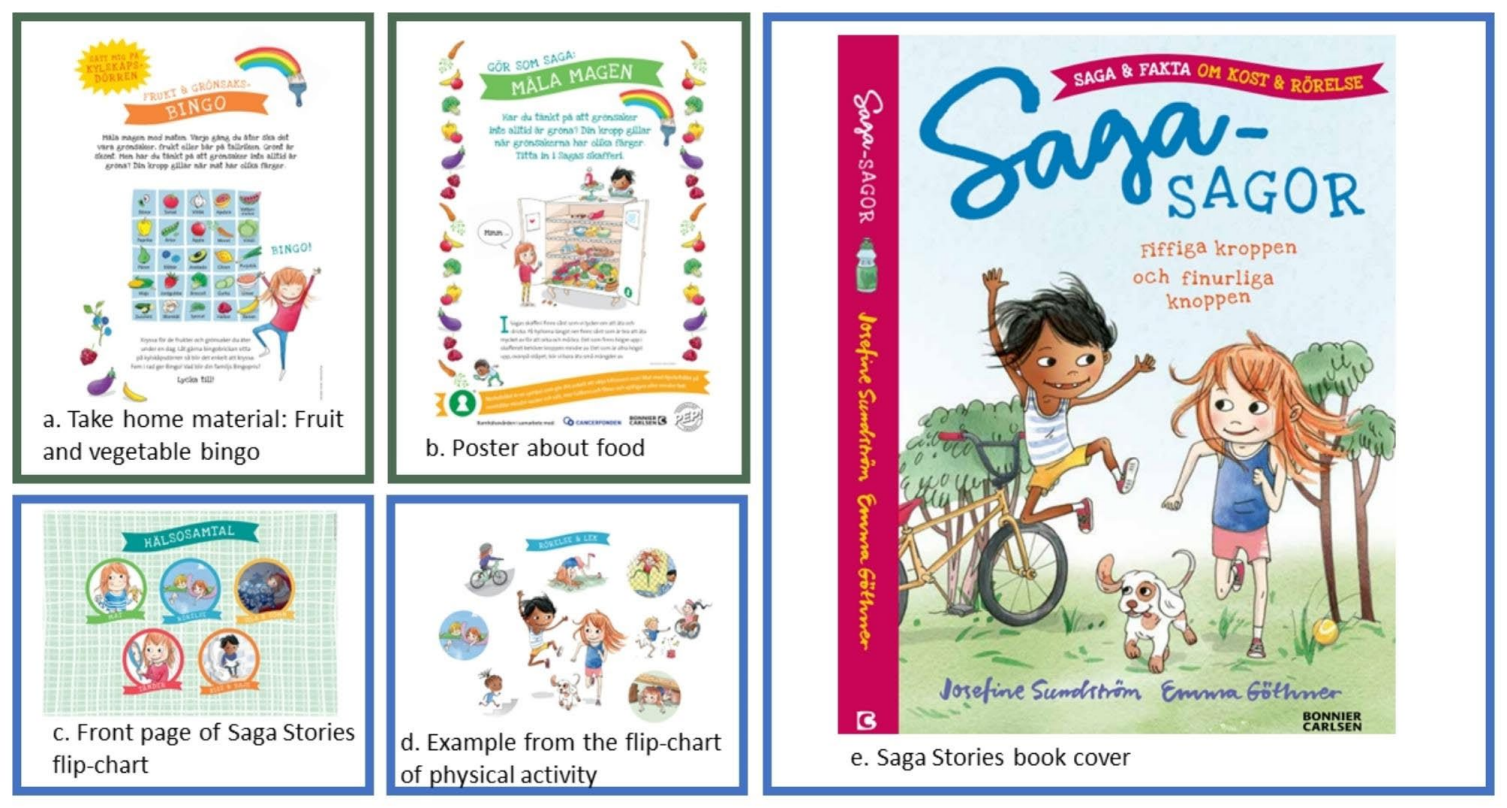
Examples of the Saga stories material

‘Saga Stories’ was initially developed as a children’s book (‘Saga Stories: Your amazing body and brain’) [20] by a non-profit organization (Generation Pep), the publisher Bonnier Carlsen, the author Josefin Sundström the illustrator Emma Göthner and experts in the areas of child health care, physiology and medicine. Generation Pep works with health promotion for children and youth in Sweden. The book was first published in 2017 and has since then been distributed to families in primary CHC during their routine visit when the child is 5 years of age. The book is intended to promote healthy lifestyle habits, including healthy diet and physical activity. Based on this book, the same developers (as described above) together with representatives from CHC centers (nurses, dieticians and pediatricians) and researchers (others than the researchers responsible for the evaluation of the material) in Sweden have developed a material for CHC nurses to use in health talks. The material was developed during 2020. The developed material **‘**Saga Stories in health talks’ includes: (1) the Saga Stories book “Saga Stories: Your amazing body and brain” [20], (2) material for the nurse to use to facilitate the health conversation with families, directed both to parents and the child at the 4- or 5-year routine visit in primary child health care, and (3) take-home material for the families to use at home. The material that is intended to be used in the health talks consists of a flip-chart with pictures and text, as well as information and questions in the areas of food, physical activity, sedentary behavior, sleep, dental health, and bathroom habits. The take-home material is handed out to promote healthy lifestyle habits in the home environment. The material includes a fruit and vegetable bingo and a physical activity fortune teller as well as a hand-out showing a 24-hour period of suggested time for sleeping, playing, being physically active, and using screens for children. See Fig. 1 for examples of the material. (insert Fig. 1 here) In this pilot study, the newly developed material ‘Saga Stories in health talks’ was pilot tested in three regions (Östergötland, Halland and Sörmland), in Sweden. The regions were selected to represent a variation in urban and rural areas as well as in different socioeconomic areas. In total 11 CHC centers and 33 CHC nurses agreed to participate in the project during the spring of 2021. The book ‘Saga Stories: Your amazing body and brain’ [20] was initially handed out at the 5-year routine visit at CHC in Sweden when the nurse has a health talk about healthy lifestyle habits with the families. In this study, the material ‘Saga Stories in health talks’ was used at the 4-year routine visit. The main reason for using the material at 4-years was to establish healthy lifestyle habits at young age. All participating CHC nurses received the material in the spring of 2021 and were invited to an educational day in February 2021 about the project and the material. The CHC nurses were then able to use the material in their health talks with families for 12 weeks before the interviews.

### Data collection

#### Recruitment

All participating CHC nurses, in the pilot project (n = 33) were invited through email to participate in an interview by the research team. The inclusion criteria were participation in the pilot study, and there were no specific exclusion criteria for the study. Of the 33 CHC nurses, 17 agreed to participate and six actively declined to participate. Reasons not to participate were a change of workplace, not being able to participate in the project due to sick leave or not using the material during the study period. Ten CHC nurses did not respond to the invitation, despite reminders. In total 17 interviews were conducted. After these interviews, saturation was achieved in the data collection, and no more reminders were sent out.

#### Participants

All participating CHC nurses were registered nurses, with a specialization in public health nursing (n = 11) or children and youth (n = 6). The CHC nurses were all females with a mean age of 42.7 years (range 35–58 years) and a mean work experience of 6.5 years, ranging from 1 to 21 years at a CHC center. They had used the material for 12 weeks, and the number of occasions varied depending on how many 4-year routine visit they had during the period for the project.

#### Interviews

The data collection consisted of individual interviews conducted in May, June, and August 2021. All interviews were conducted by telephone, due to the Covid-19 pandemic, by one of the researchers (SA). The interviewer (SA) met most of the CHC nurses at a digital introduction information meeting for the study in February 2021. The interviewer briefly presented the aim of the study and herself as a researcher and lecturer in with an interest in healthy lifestyle habits in the beginning of the information meeting. Furthermore, it was clarified that the researchers had not been part of developing the material. The information included how and when the CHC nurses would be contacted with an invitation for an interview.

In the interviews a semi-structured interview guide was used (see Appendix 2). The guide was developed and discussed by the research team and then tested in the first interview, with no correction afterwards. The interview guide consisted of overall questions about: (1) The use of **‘**Saga Stories in health talks’, (2) Perception of how the families received **‘**Saga Stories in health talks’ during the CHC visit and at home, and (3) Organizational aspects of working with **‘**Saga Stories in health talks’, including the education they had received. The interview questions were followed up with additional probes such as “Can you give an example?”, “How do you mean?”, to encourage the participants in the ongoing dialogue. Thus, the interviewer encouraged and allowed the participants to bring up new aspects and further develop areas related to the aim of the study. One interview was conducted with each participant, and field notes were taken during and after each interview.

The interviews lasted between 28 and 52 min, with a mean of 45 min, and were audio-recorded and transcribed by a professional transcriber.

### Data analysis

All data were analyzed with manifest content analysis [18, 21]. At first, the whole material, i.e., the transcribed interviews and the field notes, were read through to get an overall understanding by three researchers (SA, CC and LD). Then, each interview was coded independently by the same researchers. The coding process was first performed in word then also in excel, where meaning units were summarised into condensed meaning units. The condensed meaning units were then sorted into inductively derived sub-categories and categories. Finally, all authors critically reflected on the emerging results. The transcripts were not returned to participants for comments, and they were not asked to provide feedback on the emerging results.

Two of the authors (CC, LD) are female nurses with a specialisation in public health nursing. One of the authors, the interviewer (SA), is a female researcher and lecturer in nursing with a PhD in medical science, and extensive experience in qualitative research. The remaining co-authors (MH, CDN and ML) are nutritionists and female researchers. The interviewer and the other participating researchers had not been part of the development of the material. However, they were interested in healthy lifestyle habits, and curious about how the nurses and families would experience it in the health talks. The researchers discussed their thoughts and reflections during the whole process of data collection, analysis and reporting to recognise any pre-understandings. During this process, the researchers that preformed the analysis kept a curious mind, with an open approach to new interpretations. They also returned to the whole material in order to consider different thematic and search for negative findings, contrasting the emerging results.

### Ethical considerations

The study was approved by The Swedish Ethical Review Authority (2021 − 01764). All the methods included in this study are in accordance with the declaration of Helsinki. All CHC nurses received written and verbal information about the study and all participants gave informed consent in writing before participating in the interview.

## Results

**Fig. 2:**
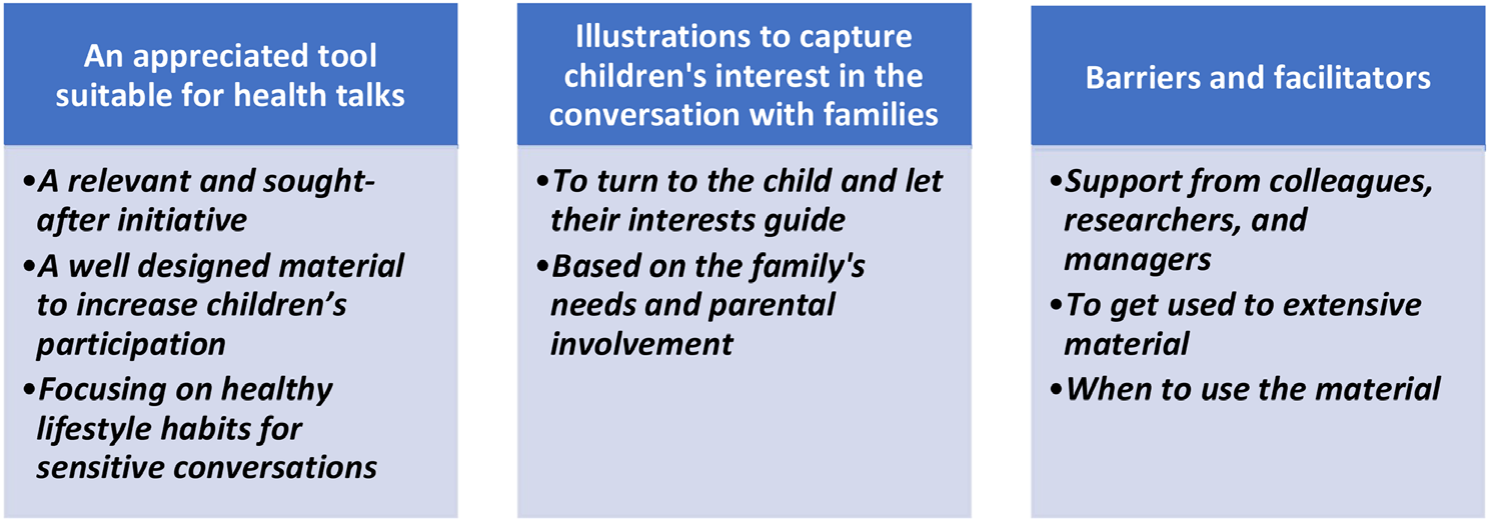
Categories and sub-categories

The findings are presented in three categories and eight sub-categories covering: *An appreciated tool suitable for health talks, Illustrations to capture children’s interest in the conversation with families*, and *Barriers and facilitators.* The categories and sub-categories are presented in Fig. 2 and in the text below.

### Category1: an appreciated tool suitable for health talks

#### Sub-category 1.1: a relevant and sought-after initiative

Being part of the project was something the CHC nurses’ thought was interesting and relevant. Many of them had worked for a long time at CHC and appreciated distributing the book ‘Saga Stories: Your amazing body and brain’ since a few years back. To get new material that was more targeted to children, for example with more child-friendly illustrations and relevant and informative topics such as support in the health conversation, was something they had wished for:

*Yes, but we thought it [the material] was really interesting because we have been able to hand out the book Saga Stories…to all 5-year-olds and… I think that has been very nice. Most children appreciate to get a book very much… So, it seemed interesting and fun, we wanted to expand this, and use the material in the health conversations. (Nurse 1)*

The CHC nurses had used different material to facilitate their health talks previously, but they emphasized that the previous material had not been as relevant to use in the health talks. The new material was in line with what they were supposed to discuss concerning healthy lifestyle habits, and thus very relevant and valuable to use in the health talks. Furthermore, the material had illustrations that helped the CHC nurses to visualize the different health aspects for the children. A nurse explained:

*Previously I have only been able to talk to the children but now [with the new material] there are nice pictures to show. So, it is also visual for the children*. *(Nurse 12)*

Some of the CHC nurses also mentioned that the newly developed material (i.e., **‘**Saga Stories in health talks’) met the needs they had identified in their respective areas, for example that there are many children with overweight. The CHC nurses emphasized that the material **‘**Saga Stories in health talks’ was a relevant and sought-after initiative to facilitate the health talks.

#### Sub-category 1.2: a well designed material to increase children’s participation

The CHC nurses’ overall impression with the **‘**Saga Stories in health talks’ material was that it has nice and fun pictures that it is well adapted for children and that it is informative with relevant topics. The importance of the child’s participation in the health conversation was something the CHC nurses constantly emphasized. They clearly described how important it is for them to involve the child in the conversation. This was also mentioned with regards to the United Nations Convention on the Rights of the Child:

*We consider how we can design the visit based on the Convention on the Rights of the Child to get the child involved. I use the picture (from the****‘****Saga Stories in health talks’ material) of what we are going to do in the visit, to explain to the child what the visit will look like today. (Nurse 1)*

Even though the CHC nurses described how they wanted to involve the child in the conversation, they emphasised that healthy lifestyle habits are the parents’ responsibility, and not the child’s.

#### Sub-category 1.3: focusing on healthy lifestyle habits for sensitive conversations

The CHC nurses described how the **‘**Saga Stories in health talks’ material contributed to making it easier to address healthy lifestyle habits in the health conversations. Important aspects were that the material has a focus on healthy lifestyle habits, with a health and health promoting focus and not on risk aspect with overweight and obesity. This was perceived as helpful for reaching out to the family. The material and the health conversation could be presented as something they offer to all families:

*I present the material as things that we all feel good about [health promoting aspects]… as something that we offer to all families. It is an easier approach, than to bring up that I suspect that a child is not moving enough. I feel good to have that role, to talk about health. (Nurse 2)*

This was specifically helpful since the CHC nurses described that it can be difficult to discuss certain topics with parents, such as when a child has gained weight or when a child is given a lot of screen time. It is a balance to talk about healthier lifestyle habits with parents, without the parent feeling stress and guilt.

### Category 2: illustrations to capture children’s interest in the conversation with families

#### Sub-category 2.1: to turn to the child and let their interests guide

The importance of capturing the child’s interest was evident in the CHC nurses’ interviews and the material facilitated this approach. The children were often described as being interested in the images and topics in the material:

*Now I manage to involve the children in a completely different way, because of the material, that it is aimed to the children. Before, we have had pictures to show to the kids, but it is not at all on the same level. This material [****‘****Saga Stories in health talks’] is more aimed directly towards children. (Nurse 3)*

The CHC nurses described how they directed the conversation directly to the child in the health conversation. They usually showed a picture with an overview of different topics in the conversation to let the child choose the first topic:

*I think that the image called ‘health conversation’ is very good to have as a starting point. In that picture, there are [small pictures of] food, physical activity, rest, sleep, you have all the categories. I then ask the children what they want to start talking about. (Nurse 4)*

The child’s interest was often allowed to guide the health conversation. The CHC nurses pointed out, that they managed to involve the child more after they started using the **‘**Saga Stories in health talks’ material.

*It’s a good thing, you notice that you capture the child’s [attention]. That we get the children engaged in a different way than before. Before, it was just a discussion with the parents. (Nurse 3)*

The book ‘Saga Stories: Your amazing body and brain’ belongs to a series of books with the same main characters, Saga and Samir. It was perceived as positive that many of the children recognised and liked these characters. There are also specific concepts in the material that were described as child friendly and useful to clarify and make the health messages understandable for the children.

#### Sub-category 2.3: based on the family’s needs and parental involvement

To enhance parental involvement and make the health conversation as relevant as possible it was important to consider the family’s needs. Sometimes the nurse knew the family and their potential needs, sometimes they asked the parents what they would like to discuss. The CHC nurses described that the parents, in general, were interested and engaged in the health conversation. The CHC nurses clearly pointed out that it is the parents that need the information but that they could reach them better by talking to the child.

*I think that it is the parent(s) that you talk to through the child. Although you maybe turn to the child, I think it’s still the parent(s) who need to get the information. (Nurse 8)*

The parent became involved, even if most of the conversation took place with the child. If the child was unfocused, silent, or did not want to participate, the nurse described how they turned more to the parents:

*But I probably turn to the child first, but when I feel that the child needs to sit and play instead, that they don’t want to talk about this anymore: Then I say a little summary or a few words to the parent. (Nurse 12)*

At the end of the visit, the CHC nurses handed out the take-home material, such as the fruit and vegetable bingo and a physical activity fortune teller to all families. The hand-out showing a 24-hour period of suggested time for sleeping, playing, being physically active, and using screens were mainly given when they thought that the family needed this type of support. The CHC nurses observed that the material, and the book ‘Saga Stories: Your amazing body and brain’ was very appreciated by the parents and that some parents also asked for more material.

### Category 3: barriers and facilitators

#### Sub-category 3.1: support from colleagues, researchers, and managers

Support from colleagues, researchers, and managers were important facilitators. Most of the CHC nurses had participated in a one-day education session with the developers and researchers and one or more follow-up meetings to be introduced and discuss the **‘**Saga Stories in health talks’ material:

*The information we have received about how to use the material and so, I think that has been good and we have had a follow-up meeting. Yes, I think that in general there has been good information about the idea with****‘****Saga Stories in health talks’ and how we should use the material in the health conversations. (Nurse 14)*

The CHC nurses described that a key facilitator was the support they experienced from their colleagues at CHC when discussing how to use the material in the health conversations. The manager’s support was also important. Some of the CHC nurses had been able to extend the time for the visit, with support from their manager.

#### Sub-category 3.2: to get used to extensive material

The **‘**Saga Stories in health talks’ material contained a lot of information covering different areas. Some CHC nurses mentioned that the material was too extensive, even if it was relevant.

*When I gather the whole material it becomes very, very big. That is probably a difficulty, which pieces to choose to focus on with each child. (Nurse 4)*

Even if some CHC nurses mentioned that the material could be simplified, most CHC nurses did not want to shorten or simplify the material. Some CHC nurses also suggested that it would be good to have even more material and information to use. Some started with the health conversation, others preferred to do the other mandatory steps of the visit first, such as checking the children’s hearing and eyesight, and take the health talk later or last during the visit. Each nurse found their own way to structure the visit and how they preferred to use the material. As the CHC nurses got more acquainted with the material, it became easier for them to use and to choose what was relevant for each family.

#### Sub-category 3.3: when to use the material

The CHC nurses stated that using the **‘**Saga Stories in health talks’ material at the 4-year routine visit was challenging, even if the CHC nurses thought that the families would benefit from having the health conversation when the child is young. One challenge with the 4-year visit was that the visit contained many other mandatory parts, such as checking the children’s hearing and eyesight. Therefore, most CHC nurses suggested to have the health conversation on the routine visit at age 5 instead. This was considered as a better option because there were fewer mandatory health checks at that time point. Some CHC nurses also thought that the children were too young to receive the information at the age of 4:

*The 4-year visit has many parts, there are fewer moments at the 5-year visit. It would fit better there, although it is good to start early. Before the age of 4, on the other hand, could be difficult, then the children are so young. (Nurse 1)*

Some of the CHC nurses also emphasised that it would be better and be easier for the child to understand and concentrate when they are 5 years old. Many CHC nurses stated that for 4-year-olds who have good linguistic ability and concentration, the conversation generally went well. However, it was more challenging if the child was tired, shy or did not want to participate.

Having a more structured health conversation could take more time, and some CHC nurses described that they had extended the time of the health conversation by 15 min. To have a separate visit for the health conversation, that focused solely on health and lifestyle, was suggested. However, they highlighted that it could also be difficult to get parents to come for an extra visit, unless they had a specific need for it.

## Discussion

This study explored the use of the newly developed material, **‘**Saga Stories in health talks’, from the CHC nurses’ perspectives. The results of this study highlights how the **‘**Saga Stories in health talks’ material helped the CHC nurses to capture the child’s interest in health talks directed to families at CHC centres. The material was found to have relevant information and to be well-adjusted with a child friendly content. Both children and their families seemed to appreciate the material according to the CHC nurses. Barriers included structural factors such as how and when to best use the material. Even if several studies [22, 23] have emphasised the importance of health promotion directed to families with small children to establish healthy lifestyle habits at a young age, there is a lack of descriptions of how to facilitate such discussions. This current study further the understanding of how CHC nurses can work with a new supportive material in health conversations.

This study shows how the material **‘**Saga Stories in health talks’ can be used to involve children in the health conversation at CHC, where the CHC nurses emphasized how they used the material to enhance the interaction with the child. In the results section, it describes how CHC nurses captured and let the child´s interests guide the health conversation using the material. Several studies have emphasized the importance of making the family feel involved in the health conversation with a committed and interested nurse, and specifically to listen to and involve the child in health care encounters [12, 24–26] as well as the importance to address health care encounters with children in relation to the UN Convention on the Rights of the Child [26, 27]. Söderbäck et al. [26] elaborate that both a child perspective, that have the children’s best interests in focus, and the child´s perspective, that acknowledge the child as an actor with respect to his or her preferences are important in health care encounters. Furthermore, Harder et al. [25] draw on the concepts of mutuality and alienation to further the understanding of health care encounters with children and parents. They state that a health care situation preformed in mutuality is performed together with the child with respect and understanding whereas an encounter in alienation lack respect and understanding. In the latter case, the health care professional does not actually encounter the child, nor do they manage to establish a relationship. The CHC nurses in the current study emphasize how the use of the material facilitated their encounters with the child where they were able to better focus on the child needs and wishes in the health talk. This approach with health talks in CHC can be understood as a prerequisite for good caring encounters.

In the current study it was highlighted that the CHC nurses main focus was on the involvement of the children in the health talks; however, we also found that the CHC nurses also wanted to increase parental involvement simultaneously. The child-centered approach was seen as an important segue to include the whole family in the health talks. The CHC nurses highlighted that the parents had the responsibility for the child´s lifestyle behaviors and thus, needed to be highly involved in the health conversation. Sometimes, the parents became involved by listening to the conversation between the child and nurse rather than the nurse having the conversation directly with the parent. This is in line with previous research regarding child-centered health dialogues in CHC [28], where a child-centered approach using illustrative material has been shown to give space for both parental and child involvement.

Furthermore, several studies have addressed that CHC nurses feel reluctant to discuss sensitive topics such as weight development [13] and screen time [22] with parents. In the present study we found that the focus on overall health and health promotion in the **‘**Saga Stories in health talks’ material, rather than targeting specific risk behaviors facilitated the discussion with the whole family. Other facilitators included the provided education to the use of the material and collegial support, that also helped the CHC nurses in how to use the material in the health talks. To further empower CHC nurses and strengthen their role in health promotion there is a need to also address structural aspects that have implications on how to preform health talks in CHC [14, 29, 30].

The main structural obstacles that emerged in this study was at which routine visit should the **‘**Saga Stories in health talks’ material be used at. In this pilot study, the material was used at the 4-year visit, which is a visit with many other mandatory components. To have the health conversation on the routine visit at age five, was considered as a better option mainly due to these structural aspects. However, the CHC nurses also highlighted that the children would be able to understand and concentrate better at five years of age. This has also been addressed by Derwig at al [31]. who showed that even though 4-year-old children were able to actively participate and to understand the meaning of health information when participating in child-centered health dialogues, they also understood the health messages differently than intended.

Previous research has shown that nurses pay infrequent attention to dietary and physical activity behaviours in children at CHC centres [16]. Considering that overweight and obesity is a growing concern in young children [1], and a sensitive topic with many barriers in health conversations [30, 32]. From this perspective, there is a need for new and innovative tools, such as **‘**Saga Stories in health talks’, to be used in these conversations. Furthermore, to have time for the discussion was considered a key facilitating factor.

### Methodological considerations

A strength with the data collection was that the CHC nurses represented all three regions that had participated in the study, and a variety of CHC centres in these regions. Furthermore, the CHC nurses represented varying work experience and were in different ages. A limitation was that some CHC nurses declined to participate. The main reason for declining to participate in the interview were time limitations.

All interviews were rich and contributed to data saturation before data collection was completed. Data was collected through telephone interviews due to the Covid-19 pandemic. It is possible that a face-to-face interview would have provided a more nuanced picture of the CHC nurses’ experiences of the material. A strength in the process of analysis was the researcher triangulation, with three researchers conducting the content analysis.

## Conclusions

This study explored CHC nurses’ experiences of the usability of the ‘Saga Stories in health talks’ material. Overall, the CHC nurses found the material, including the illustrations, to be an appreciated and suitable tool for health talks. The CHC nurses emphasised how the material helped them both to capture the child’s interest and to involve the parents in the health talks. The focus on healthy lifestyle habits in the material specifically facilitated the conversation with parents. A challenge with the conversation was to conduct the health conversation during the 4-year routine visit, since the visit had many other mandatory health checks, and since some children couldn’t concentrate on the conversation. To use the material at the 5-year routine visit could be a better option. Thus, more research is needed to explore implementation of the material in CHC in 5-year children. The next step is to evaluate the effectiveness of the “Saga Stories in health talks” on parental self-efficacy to promote healthy lifestyle behaviors in their children as well as implementation outcomes within CHC using a hybrid type 1 effectiveness-implementation design [33].

### Electronic supplementary material

Below is the link to the electronic supplementary material.

Supplementary Material 1

Supplementary Material 2

## Data Availability

The transcripts generated and analysed during the current study are not available to maintain participant confidentiality, but further information such as documentation of the analysis are available from the corresponding author on reasonable request.

## Abbreviations

CHC: Child health care
PA: Physical activity
