# Supplementary material for: To capture the child’s interest - nurses experiences of ‘Saga stories in health talks’
Source: BMC Nurs. 2024 Jan 2;23:8. doi: 10.1186/s12912-023-01661-7 (PMC10759488; doi:10.1186/s12912-023-01661-7)
Supplement: Supplementary file 2 — Supplementary Material 2 [file 12912_2023_1661_MOESM2_ESM.docx]

# Appendix 2: Interview guide

1. **Introductory questions**

How did you first come in contact with the **‘**Saga Stories in health talks’ project?

How did you feel that the project worked in general?

1. **Use of ‘Saga Stories in health talks’**

During the project, how have you used the **‘**Saga Stories in health talks’ material?

What opportunities do you see with the use of **‘**Saga Stories in health talks’?

What obstacles/difficulties do you see with the use of **‘**Saga Stories in health talks’?

1. **Perception of how the families received ‘Saga Stories in health talks’ during the visit and at home**

How do you experience the health talk with the parents and with the children?

Can you describe how the families received the health talks?

How did you work with the take-home material (the fruit and vegetable bingo, the physical activity fortune teller and the hand-out showing a 24-hour period)?

Have you used the material at any other time than the 4-year visit?

When it comes to the concepts in the material, e.g. paint the stomach, (the pantry) and the ‘movement buddies’. What are your thoughts about them?

Can you tell us about your impression of the parents' work with the material from **‘**Saga Stories in health talks’ at home?

1. **Organizational aspects of working with ‘Saga Stories in health talks’, including the training**

How did you experience the education about Saga Stories before the start of the project (content, relevance, time, suggestions for improvement)?

How did it work to work with **‘**Saga Stories in health talks’ within the framework of your work (time for conversation, relevance in the health conversation, etc.)?

What support do you think you need as a CHC nurse to work with **‘**Saga Stories in health talks’?

Do you have any suggestions on how the material can be developed?

1. **Conclusion**

Is there anything else you want to tell me about?

How have you experienced this conversation?

Thank you for your participation!
